# Molecular Characterization of the Highest Risk Adult Patients With Acute Myeloid Leukemia (AML) Through Multi-Omics Clustering

**DOI:** 10.3389/fgene.2021.777094

**Published:** 2021-10-29

**Authors:** Trinh Nguyen, John W Pepper, Cu Nguyen, Yu Fan, Ying Hu, Qingrong Chen, Chunhua Yan, Daoud Meerzaman

**Affiliations:** ^1^ Center for Biomedical Informatics and Information Technology, National Cancer Institute, Rockville, MD, United States; ^2^ Division of Cancer Prevention, National Cancer Institute, Rockville, MD, United States

**Keywords:** multi-omics, unsupervised clustering, intrinsic subtypes, acute myeloid leukemia, targeted therapeutics

## Abstract

**Background:** Acute myeloid leukemia (AML) is a clinically heterogeneous group of cancers. While some patients respond well to chemotherapy, we describe here a subgroup with distinct molecular features that has very poor prognosis under chemotherapy. The classification of AML relies substantially on cytogenetics, but most cytogenetic abnormalities do not offer targets for development of targeted therapeutics. Therefore, it is important to create a detailed molecular characterization of the subgroup most in need of new targeted therapeutics.

**Methods:** We used a multi-omics approach to identify a molecular subgroup with the worst response to chemotherapy, and to identify promising drug targets specifically for this AML subgroup.

**Results:** Multi-omics clustering analysis resulted in three primary clusters among 166 AML adult cancer cases in TCGA data. One of these clusters, which we label as the high-risk molecular subgroup (HRMS), consisted of cases that responded very poorly to standard chemotherapy, with only about 10% survival to 2 years. The gene *TP53* was mutated in most cases in this subgroup but not in all of them. The top six genes over-expressed in the HRMS subgroup included *E2F4, CD34, CD109, MN1, MMLT3,* and *CD200*. Multi-omics pathway analysis using RNA and CNA expression data identified in the HRMS subgroup over-activated pathways related to immune function, cell proliferation, and DNA damage.

**Conclusion:** A distinct subgroup of AML patients are not successfully treated with chemotherapy, and urgently need targeted therapeutics based on the molecular features of this subgroup. Potential drug targets include over-expressed genes *E2F4*, and *MN1*, as well as mutations in *TP53,* and several over-activated molecular pathways.

## Introduction

AML not only represents one of the most fatal leukemias but also ranks among the deadliest of all cancers. It presents a myriad of chromosomal alterations and gene mutations, comprising a clinically heterogeneous group of diseases (Green and Konig, 2020). Cytogenetic abnormalities (chromosomal translocations, deletions, etc.) are found in most AML cases, and strongly correlate with prognosis. Therefore, the modern WHO classification of AML categories and subtypes relies substantially on cytogenetics (Carter et al., 2020). However, unlike molecular features (mutations, overexpressed signaling pathways, etc.) cytogenetic abnormalities usually do not offer molecular targets that allow for development of targeted therapeutics. Therefore, even where cytogenetic aberrations are established and correlated with prognosis, it is important to create a detailed molecular characterization of those subtypes most in need of new targeted therapeutics. Many AML patients are treated using untargeted chemotherapy. This is effective against those cytogenetic subgroups recognized as having good prognosis under that treatment regimen, but chemotherapy offers very low survival rates to those cytogenetic subgroups recognized as having poor prognosis under this treatment regimen, with only about 20% survival beyond 2 years (Cancer Genome Atlas Research et al., 2013). Currently, nine agents have been approved, including FLT3, IDH, Bcl-2 inhibitor, and others. Due to the heterogeneity of AML, there is a need to identifying new molecular targets for future targeted therapies (Kantarjian et al., 2021). In recent years, developments in multi-omics data integration have been useful in identifying new subgroups as well as biomarkers for different types of cancers. Nguyen et al. (2020a), used three-omics profiles, DNA copy number aberration, methylation, and mRNA expression, to discover two biologically distinct subgroups in breast cancer. Zheng et al. (2020), used methylation array data and gene expression data to identify prognostic biomarkers in AML. Nguyen et al. (2020b), used mRNA, Methylation, and miRNA from many types of cancer to develop tools and discover disease subtypes. Therefore, we examined multi-omics data to seek intrinsic molecular subgroups that could guide the development of additional effective targeted therapies for patients with poor prognosis under chemotherapy.

## Materials and Methods

We began with an unsupervised clustering analysis using two types of data: somatic copy number alteration (CNA), and gene expression levels from RNA-seq measurements. We then identified differences among the three resulting clusters in their risk stratification, and in overall survival, using datasets with information on mutations and putative copy number alterations from GISTIC (Genomic Identification of Significant Targets in Cancer), with matched clinical data. Next, we performed pathway analyses to find differences among the three molecular subgroups in which molecular pathways they were enriched in. Further analyses focused on molecular characterization of the one cluster with the worst prognosis under the chemotherapy.

### Dataset Preparation

We downloaded the TCGA adult AML datasets directly from cBioPortal for cancer genomics (https://www.cbioportal.org/study/clinicalData?id=laml_tcga_pub) (Cerami et al., 2012). We used the total of 166 samples with transcriptomic, copy number alteration, mutation, and clinical datasets. These samples were obtained from peripheral blood and represented the major morphologic and cytogenetic subgroups of AML (Cancer Genome Atlas Research et al., 2013). We used two different CNA datasets: CNA segmentation and discrete CNA values datasets. For the CNA segmentation, we estimated gene level CNA as the segment mean of copy numbers of the genomic region of a gene by using TCGA-Assembler 2 (Wei et al., 2018) downloaded from https://github.com/compgenome365/TCGA-Assembler-2 (version 2.0.6). Degree of CNA was calculated as log2 (tumor values/normal values). Across samples, CNA of all genes had a standard deviation greater than the median. Therefore, to exclude near normal (very low) CNA values, only genes with a sum of CNA values across samples greater than zero were used for analysis, resulting in 13,019 genes total. Hg19 annotation was used to obtain gene position. For the integration with this CNA expression dataset, we used RSEM (RNA-Seq by Expectation Maximization) expected raw count expression dataset. Genes without at least one count-per-million reads in at least 50% of the total samples were filtered out. The resulting RNA dataset was log2 transformed and quantile normalized. A total of 12,934 genes were retained for analysis. From the discrete CNA values dataset, putative copy-number calls determined using GISTIC 2.0 were used to obtain the information. Patients with CNA values greater than or equal to 1 were classified as copy number amplifications, while patients with values less than or equal to -1 were classified as copy number deletions. Patients with zero values were classified as unchanged. We also used mutation information, to identify differences among our subgroups, focusing on genes known to be important in AML: *RUNX1*, *RUNX1T1*, *CEBPA*, *NPM1*, *DNMT3A*, and *TP53*, as well as the genes coding for the targets of currently approved targeted drugs for AML: *IDH1*, *IDH2*, *CD33*, *BCL2*, and *FLT3* (Kantarjian et al., 2021). The clinical dataset provided information on cytogenetic abnormalities and on clinical outcomes. Clinical information on cytogenetic risk and genetic abnormalities is summarized in Supplementary Table 1.

### Pathway Database

To study molecular pathways, we downloaded the gmt file of MSigDB hallmark gene set collection (version 7.1) from https://www.gsea-msigdb.org/gsea/msigdb/collections.jsp for annotation. The 50 hallmark pathways in this collection each represent a biological state or process (Liberzon et al., 2015).

### Multiple Omics Data Integrative Clustering and Gene Set Analysis (MOGSA)

**FIGURE 1:**
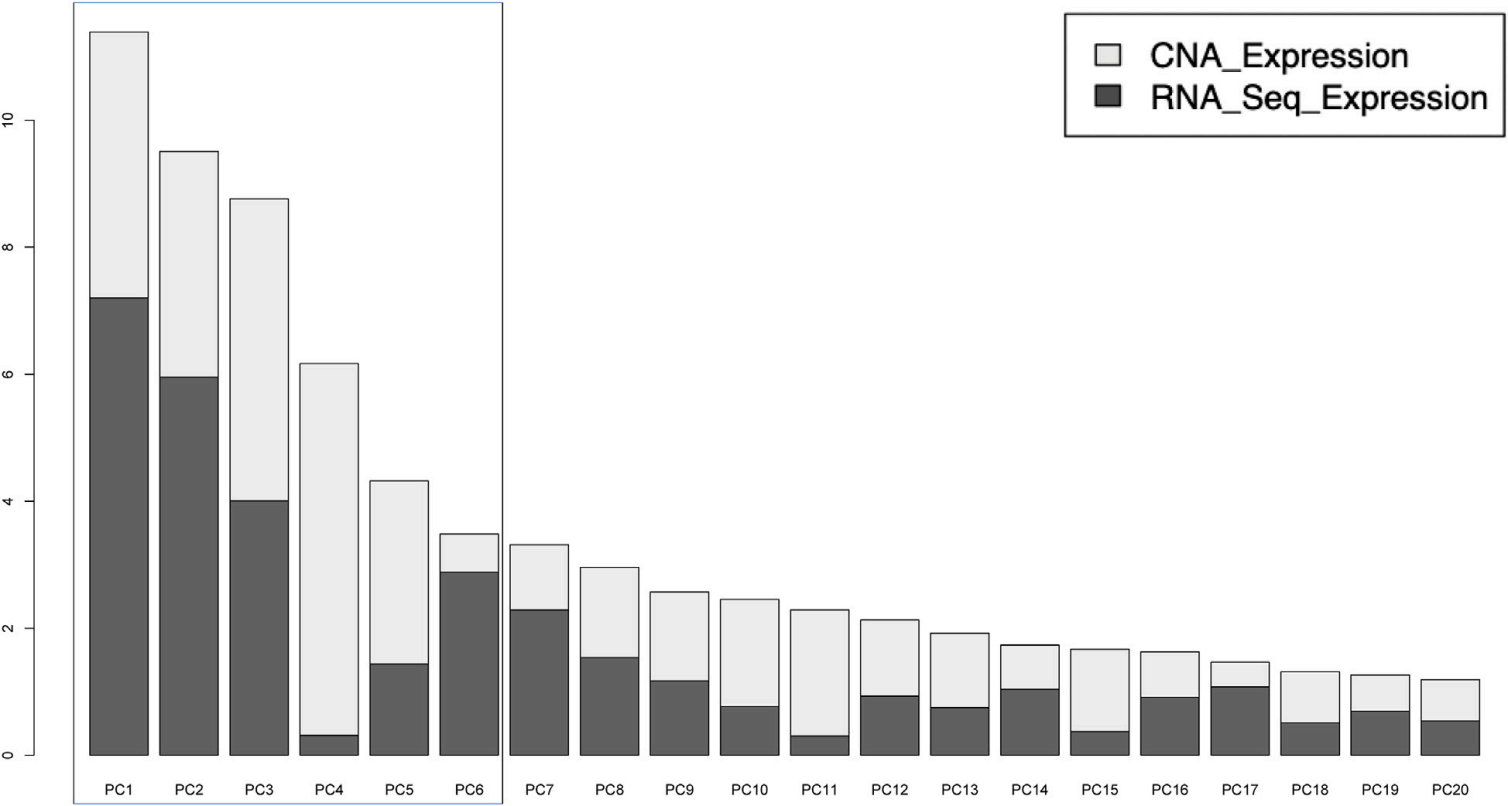
Distribution of variances explained by the 20 principal components (PCs). The first six PCs were used to identify subgroups by clustering. These contained a total of 43.6% of total variance, with CNA and Gene expression contributing equally.

MOGSA is an R software package for multivariate single sample gene set analysis (Meng et al., 2019). Using this package (version 1.22.1), we integrated transcriptomic data and gene level copy number alterations (CNA) over the same set of samples. Firstly, we performed multiple factorial analysis (MFA) (Wei et al., 2018) from MOA function of MOGSA to determine the number of principle components based on the integration of CNA and RNA-seq Expression. Next, we used the MOGSA (the Integrative Single Sample Gene-set Analysis of Multiple Omics Data) function to identify the MSigDB hallmark pathways’ gene set scores (GSS). We used these parameter settings: nf = 6 (6 chosen PCs), proc. row = ” center_ssq1″, w. data = “lambda1”, and statis = FALSE. In order to recognize potential intrinsic subgroups among the cases, we used ConsensusClusterPlus (version 1.52.0) (Wilkerson and Hayes, 2010) to identify clusters. We used correlation between variables from the first 6 PCs derived from MFA (Figure 1) as the distance, and with these parameter settings: maxK = 6, reps = 10,000, pItem = 0.8, clusterAlg = ” hc”, finalLinkage = “ward.D2”, distance = “pearson”. Lastly, to choose representative molecular pathways from the selected three clusters, we selected the pathways resulting from the MOGSA function with GSS FDR (false discovery rate) values smaller than 0.01 in 50% of all samples. We used the R functions, fitting generalized linear models (GLM) to calculate the difference of GSSs in each subgroup versus that in the rest and selected the top five and bottom five representative pathways ranked by GLM T values, resulting in 16 unique representative pathways with GLM FDR <0.01. The three subgroups differ significantly in these representative pathways with ANOVA test FDR <0.001. We visualized z-score scaled median GSSs in a heatmap to show the overall pathway enrichment from both data types as well as the contribution of each data type to the subgroups (Figure 3B).

### Survival Analysis

We used the R modules Survfit and coxph (Therneau and Grambsch, 2000) to perform overall survival analysis based on the three subgroups resulting from the total of 166 TCGA adult samples.

## Results

From the MFA analysis, the first 6 PCs were chosen for unsupervised clustering gene set analysis due to the equal contribution of CNA and RNA-seq expression (Figure 1)

**FIGURE 2:**
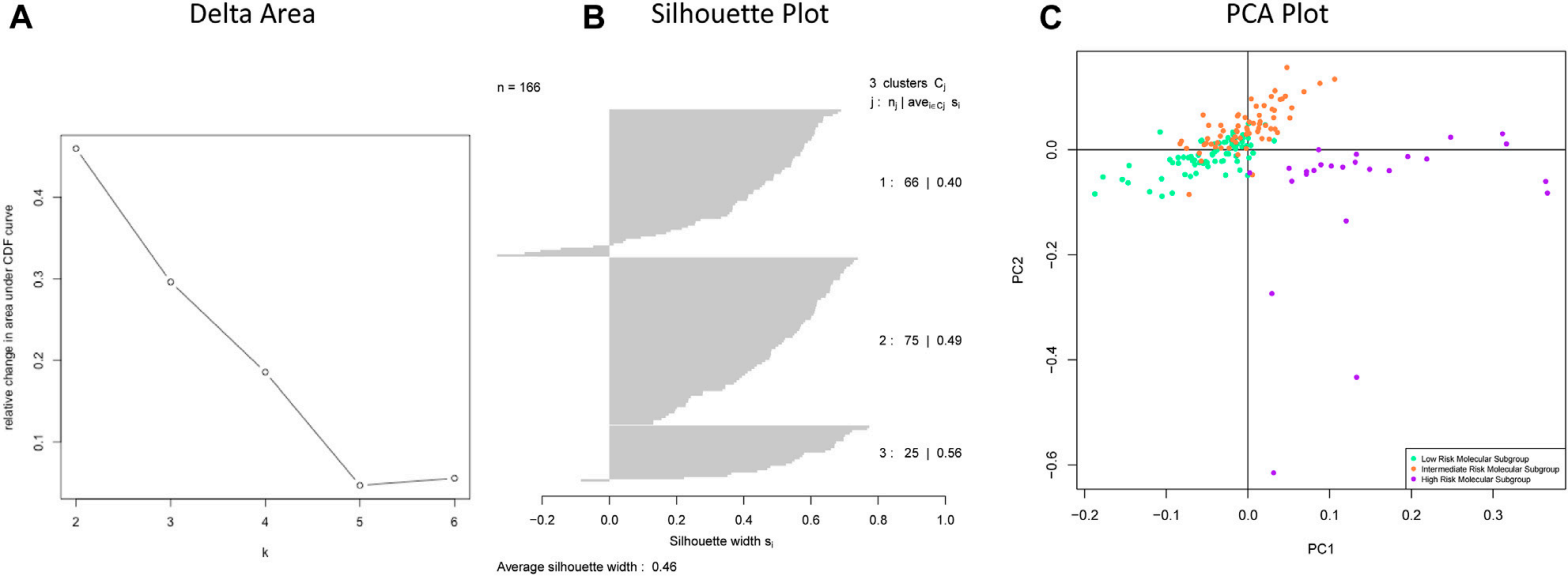
**(A)** Delta area shows the numbers of clusters (k) (*X* axis) and their relative change in area under CDF curve (*Y*-axis). **(B)** Silhouette plot of chosen clusters with k = 3. **(C)** The separation of three subgroups: Low Risk Molecular Subgroup, Intermediate Risk Molecular Subgroup, and High-Risk Molecular Subgroup.

**FIGURE 3:**
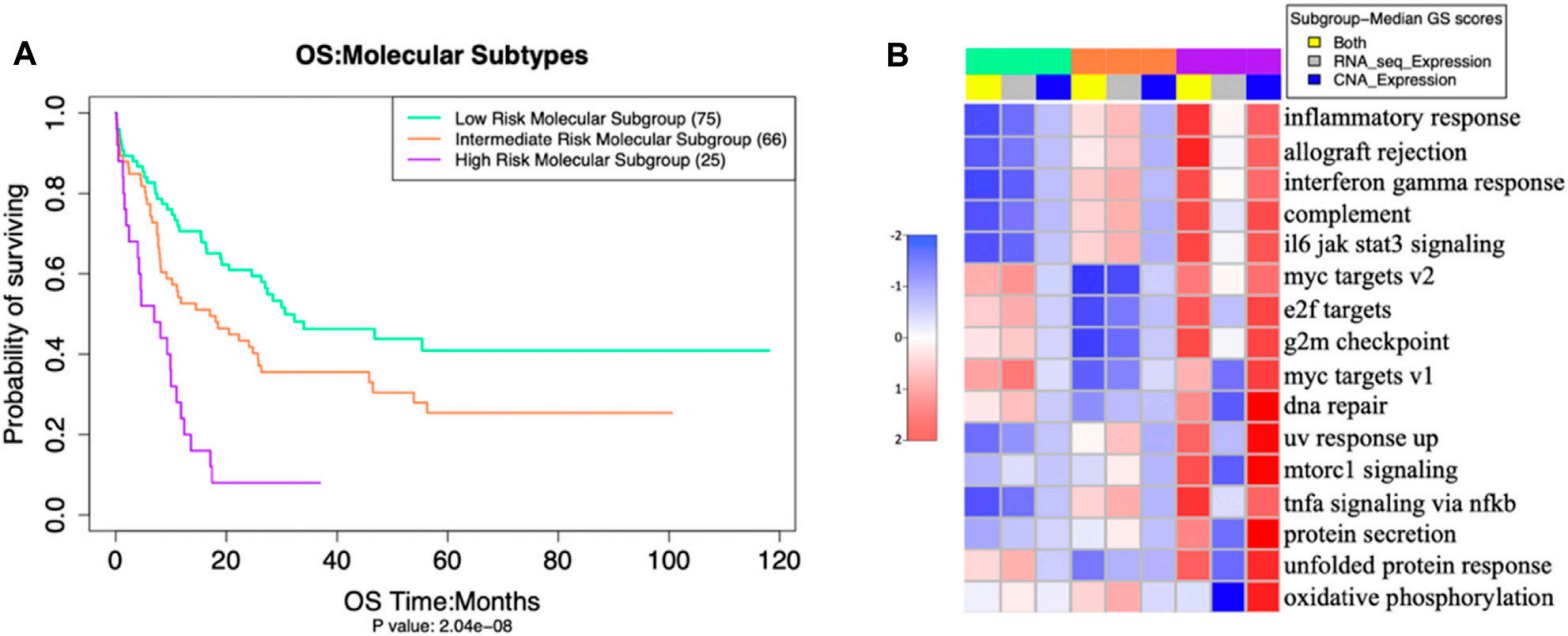
**(A)** Overall Survival outcomes of our three molecular subgroups differed significantly (*p* = 2 × 10^−8^ (adjusted by gender and age) **(B)** activation levels of 16 pathways (in rows), also differed significantly, as measured by Multi omics Gene set Scores analysis of molecular subgroups with ANOVA test -- false discovery rate (FDR< 0.001).

Through unsupervised clustering, we selected the three subgroups as the best clustering solution because this number of clusters gave the greatest area under the CDF curve (Figure 2A), and the best separation of clusters (Figures 2B,C). We named the three resulting clusters as follows: C1 or “Intermediate Risk Molecular Subgroup”; C2 or “Low Risk Molecular Subgroup”; and C3 or “High Risk Molecular Subgroup”. Descriptive names were based on our survival analysis (Figure 3A).

The three putative subgroups of cases resulting from our unsupervised clustering analysis (Figure 2) differed from each other both in their prognosis (Figure 3A), with *p* = 2 x 10^−8^ (adjusted by gender and age), and in their molecular traits, with ANOVA test FDR <0.001 (Figure 3B).

**FIGURE 4:**
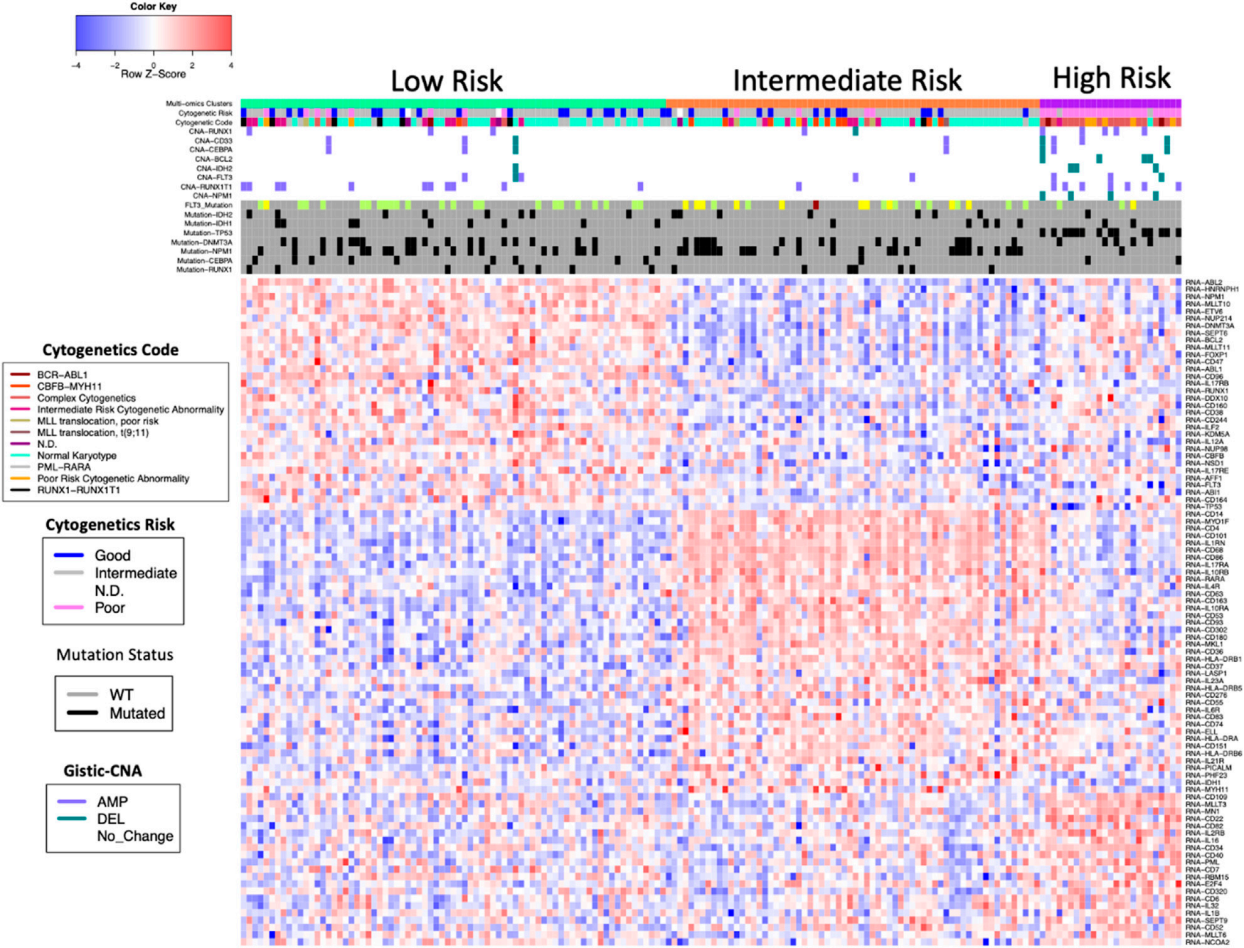
Summary of molecular differences among the three patient subgroups.

These three putative AML subgroups also differed in several other aspects of their molecular makeup (Figure 4).

Among the eleven AML genes we examined, the HRMS subgroup had significantly fewer gene mutations than the other patients. In contrast, among these eleven AML genes, the HRMS subgroup had a higher frequency of copy number alterations (CNAs) (Figure 4). When separated by CNA type, this difference was statistically significant for copy-number amplifications (Fisher exact test, *p* = 0.014), and for copy-number deletions (*p* = 0.0001).

**FIGURE 5:**
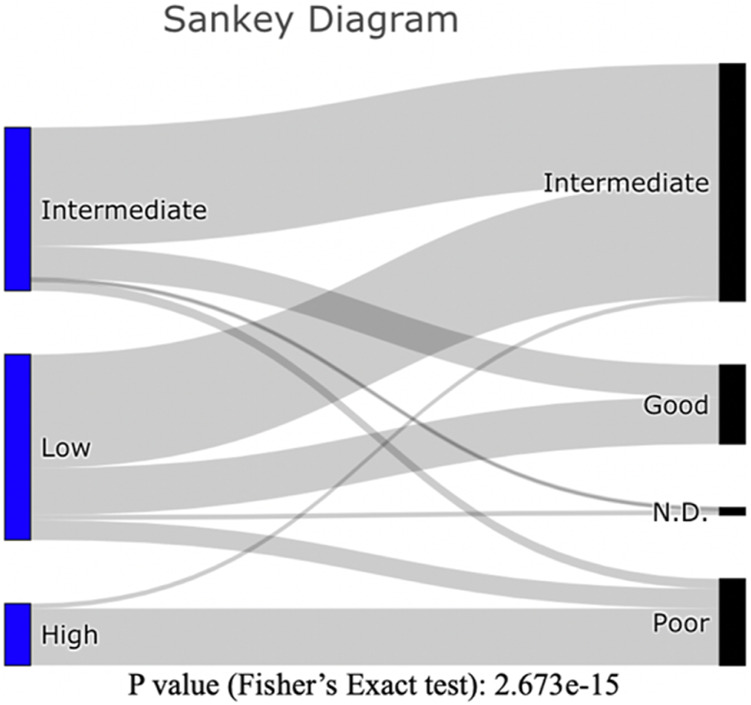
Sankey Diagram: Counts of patients in our three molecular subgroups (Left), as classified by established cytogenetic risk levels (Right). Counts of patients can be found in Supplementary Table 2.

The patients in our HRMS subgroup had significantly lower overall survival than did other patients (Figure 3A). This was largely consistent with their risk stratification based on cytogenetics (see Supplementary Table 2). The three new molecular subgroups were significantly associated with established cytogenetic risk stratifications from clinical data (Figure 5; Fisher’s exact test, *p* value <10^−14^). Among our samples, most patients with a “poor” cytogenetic risk classification fell within our multi-omics HRMS, while all those with a “good” cytogenetic risk classification fell into other subgroups. As expected, based on this association with established poor cytogenetic risk stratification, HRMS patients had poor overall survival. However, HRMS included only a subset of the poor cytogenetic risk group patients in our dataset (23 of 35 total, see Supplementary Table 2), and this subset had even worse survival than did cytogenetic poor risk patients as a whole set.

In a previous analysis, overall survival of patients in the poor cytogenetic risk group at 2 years was reportedly about 20% (Cancer Genome Atlas Research et al., 2013) (we replicated this result with our subset of 166 of the 200 patients used in the earlier study, P (adjusted by gender and age) = 9E-09, see Supplementary Figure 1). In contrast, among the patients in our HRMS subgroup, overall survival at 2 years was much worse, at only about 10% (Figure 3A).

Hereafter, we focused on the high-risk molecular subgroup (HRMS), because patients in this subgroup had significantly worse clinical outcomes than other patients and did not over-express the drug-target genes for existing targeted therapeutics (*IDH1*, *IDH2*, *CD33*, *BCL2*, and *FLT3*). We focused on this molecular subgroup for further molecular characterization in search of promising new drug targets.

The multi-omics pathway analysis using RNA and CNA expression data revealed significant differences among the molecular subgroups in the combined activation gene set scores of various molecular pathways from both datasets (see Supplementary Table 3 for the single gene set scores (GSS) of these pathways). The HRMS subgroup showed higher activation than other patients of most molecular pathways related to immune function, cell proliferation, and DNA damage, with CNA expression contributing more than RNA-seq expression to this overall GS (Figure 3B).

Among genes known to be important in AML, mutation frequencies differed in our HRMS subgroup versus other patients (Table 1). Among the nine AML genes in our mutation data set, most (6/9) had lower mutation frequencies in HRMS than in other patients, but these differences were not statistically significant. In contrast to the other AML genes, *TP53* was mutated in most patients in the HRMS, but not in any other patients, constituting a highly significant difference (Table 1).

**TABLE 1:** Frequencies of mutation in AML genes in HRMS subgroup, versus other patients. *p*-values are from Fisher’s exact tests on counts of mutant and wild-type genes.

Gene	Frequency in HRMS (%)	Frequency outside HRMS	*p* value (<0.01)
*RUNX1*	0	11%	NS
*RUNX1T1*	0	1.4%	NS
*CEBPA*	8	7.1%	NS
*FLT3*	12	31%	NS
*NPM1*	4	33%	0.003^a^
*DNMT3A*	20	24%	NS
*TP53*	56	0	10^−10^ ^a^
*IDH1*	8	9%	NS
*IDH2*	4	10.6%	NS

Note: NS: nonsignificant, and.

a significant.

### Gene Over-expression

The dataset for RNA-seq included 11 genes known to be important in AML: *IDH1*, *IDH2, CD33, BCL2, FLT3, DNMT3A, NPM1, CEPBA, RUNX1, E2F4,* and *TP53*.

**FIGURE 6:**
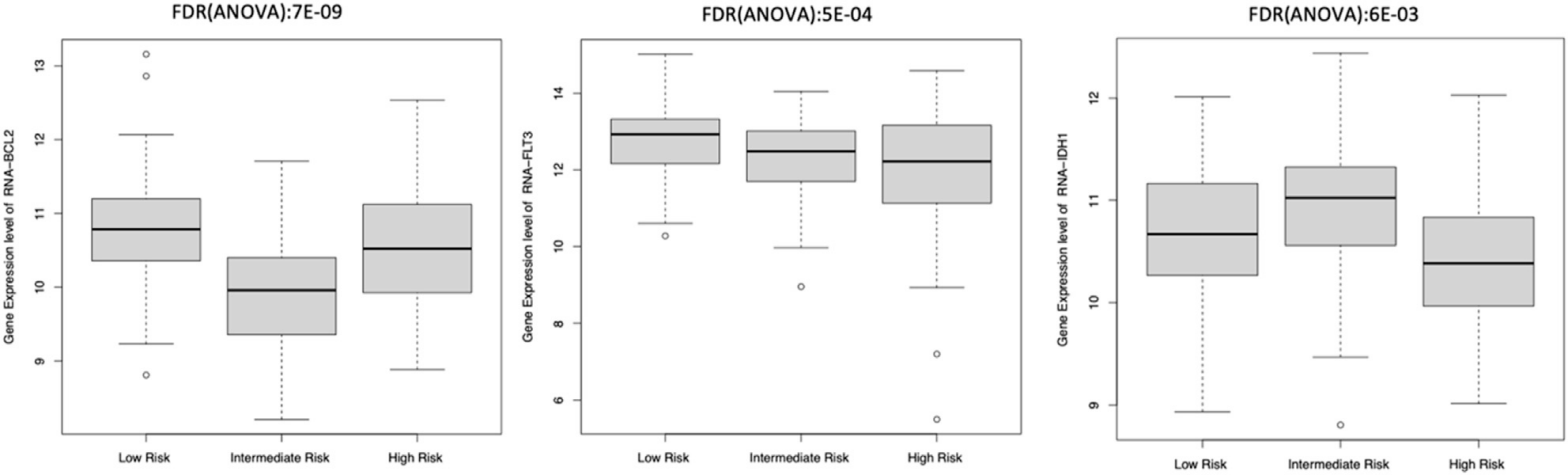
*BCL2, FLT3, and IDH1* gene expression.

**FIGURE 7:**
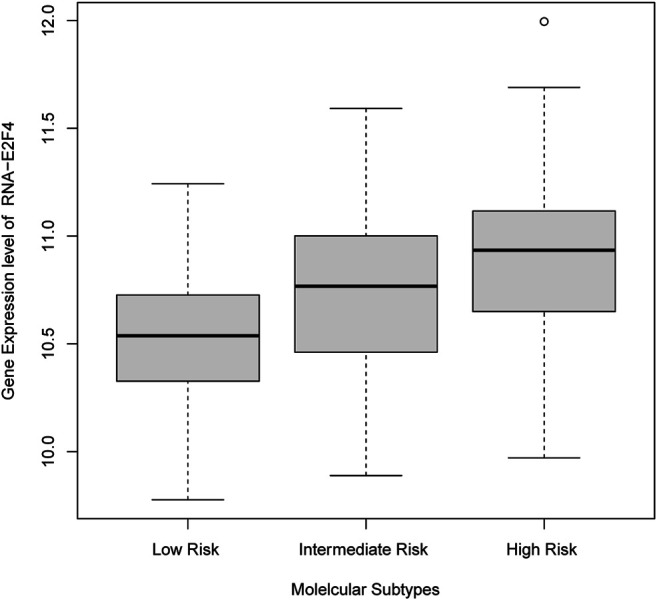
*E2F4* gene expression was elevated in the High-Risk Molecular Subgroup.

Eight of these 11 genes varied significantly among clusters (ANOVA test, FDR <0.01). The other three genes, including *IDH2* and *CD33* inhibitors, did not differ significantly among these subgroups. We observed that *BCL2* and *FLT3* were elevated in Low-Risk while *IDH1* was elevated in the Intermediate-Risk subgroup (Figure 6). Only one of these genes, *E2F4*, had elevated expression in the HRMS subgroup (Figure 7). This difference was highly significant (ANOVA FDR = 8E-06).

In addition to the eleven genes listed above, we compiled expression data on a total of 135 AML genes involved in rearrangement, immune interaction, and blast from Mitelman database, OMIM, and publications (see Supplementary Table 4, “146 analyzed AML genes”). Altogether, we identified a total of 104 genes that differed significantly among these three molecular subgroups with Anova FDR <0.01 (see Supplementary Table 5, “significant AML genes”). We further looked for genes that were more highly expressed in one subgroup and found that 32 genes were highly expressed in low Risk, 39 genes were highly expressed in intermediate risk group, and 21 genes were highly expressed in high risk (Figure 4 and see Supplementary Table 6, “significant AML genes in subgroups”).

## Discussion

To shed light on whether currently available drugs might be well suited for each of our molecular subgroups, we examined each subgroup for expression levels of the drug-targeted genes: *BCL2* (venetoclax), *FLT3* (midostaurin and gilteritinib), *IDH1*, and *IDH2* (enasidenib and ivosidenib), and *CD33* (gemtuzumab and ozogamicin) (Carter et al., 2020; Kantarjian et al., 2021). For each of these drug targets, one subgroup showed higher expression than the others. However, among the three subgroups, the HRMS subgroup did not show the highest expression of any available drug target (Figure 6). This suggests a need for new potential drug targets for this subgroup especially. The HMRS subgroup that we identify here based on molecular markers, overlaps substantially with the long-established cytogenetic high-risk subtype (Figure 5), but differs in two important ways. Firstly, after treatment with chemotherapy, The HMRS had even lower survival than does the cytogenetic high-risk subtype. Thus, our molecular subgroup offers a more focused classification of cases that are not successfully treated with chemotherapy, and that therefore urgently need new targeted therapeutics. Secondly, unlike cytogenetic features, which do not offer drug targets, this subgroup is characterized by molecular traits that do offer potential as new drug targets. Our findings indicate several candidates for drug targets specific to the extremely high-risk patients of our HRMS subgroup. These candidate targets include mutations of gene TP53, which was mutated in most HRMS patients (Table 1), as well as overexpression of six genes that were highly over-expressed in the HRMS subgroup, including *CD34*, *CD109*, *CD200*, *E2F4*, *MN1*, and *MLLT3*. Other potential targets may be found in the molecular pathways that are highly activated in our HRMS subgroup (Figure 3B).

One of the strongest molecular associations with our HRMS subgroup was mutations in *TP53*. This is consistent with the fact that *TP53* mutations are known to be associated with cytogenetic abnormalities, and with poor outcomes, as is our HRMS subgroup. It has long been established that *TP53* mutations are associated with resistance to chemotherapy and short survival in hematologic malignancies (Wattel et al., 1994). The importance of *TP53* mutations specifically for our HRMS subgroup is also consistent with the guidelines of the National Comprehensive Cancer Network, which classify AML patients with normal cytogenetics into the poor/adverse risk category if they harbor *TP53* mutations (Daver et al., 2020). In AML, mutations in *TP53* are associated with poor responses to chemotherapy, and with very poor prognosis (Wang et al., 2020). These authors (Wang et al., 2020) suggested that it was important to test whether other pathways activated by *TP53* mutations could be therapeutically targeted. Our results should contribute to reaching that goal.

The overexpression of MN1 is known to confer resistance to chemotherapy, and a worse AML prognosis. Pardee (Pardee, 2012) investigated the mechanisms for this and suggested that therapies directed at increasing *TP53* function may be useful for such patients. Another of the genes most over-expressed in our HRMS subgroup was *E2F4*. This is unsurprising, as it is known that *TP53* mutations can drive the expression of *E2F4* (Blandino and Di Agostino, 2018). The over-expression of *E2F4* in our HRMS subgroup was also consistent with a recent report that *E2F4* over-expression was associated with poor prognosis in AML patients, and that in a mouse model, depleting *E2F4* inhibited proliferation and suppressed the growth of AML cells (Feng et al., 2020). These authors suggested *E2F4* as a potential therapeutic target (Feng et al., 2020), and here we support that suggestion by showing the importance of this gene specifically in the HRMS subgroup of patients expected to fare worst under untargeted chemotherapy.

Other molecular characteristics of our HRMS subgroup include highly activated molecular pathways in the categories of immune function, DNA damage, and cell proliferation, all three of which are consistent with previous reports. A high level of DNA damage has been reported for cells of AML patients categorized as having high-risk cytogenetics and is accompanied by activation of DNA damage pathway (Cavelier et al., 2009). Our results show that inflammatory response and IL6 JAK STAT signaling pathways were highly activated in HRMS. This is consistent with the findings that the inflammatory pathway leads to an activation of the JAK/STAT signaling in AML which fosters leukemia proliferation (Habbel et al., 2020).

Our results suggest that pathways activated by mutations in *TP53* might be targeted therapeutically. We found that the pathways highly activated in our HRMS are in the proliferation category, including, E2F targets, G2M checkpoint, and Myc targets V2 (see Supplementary Figure 2). Activation of these proliferation pathways can be promoted by the overexpression of the *E2F4* gene.

Limitation: Our sources provided data on a relatively small sample of cases representing the HRMS subgroup, comprising only, 25 out of 166 cases, which may limit the power of our statistical results, but is unlikely to affect the nature of the qualitative results.

## Conclusion

A distinct subgroup of AML patients is not successfully treated with chemotherapy, and urgently needs targeted therapeutics. Potential new drug targets for this subgroup include over-expressed genes *E2F4*, and *MN1*, as well as mutations in *TP53*, and over-activation of several molecular pathways.

## Data Availability

The datasets presented in this study can be found in online repositories. The names of the repository/repositories and accession number(s) can be found in the article/Supplementary Material.

## Abbreviations

AML, acute myeloid leukemia; TCGA, the cancer genome atlas; CNA, copy number alteration; RNA-Seq, transcriptomics sequencing; RSEM, RNA-Seq by Expectation Maximization; MOGSA, multiple omics data integrative clustering and gene set analysis; MFA, multiple factorial analysis; PCA, principal component analysis; GSS, gene set scores; FDR, false discovery rate; HRMS, High Risk Molecular Subgroup.
