# Supplementary figures and images for: Molecular Characterization of the Highest Risk Adult Patients With Acute Myeloid Leukemia (AML) Through Multi-Omics Clustering
Source: Front Genet. 2021 Oct 29;12:777094. doi: 10.3389/fgene.2021.777094 (PMC8585788; doi:10.3389/fgene.2021.777094)

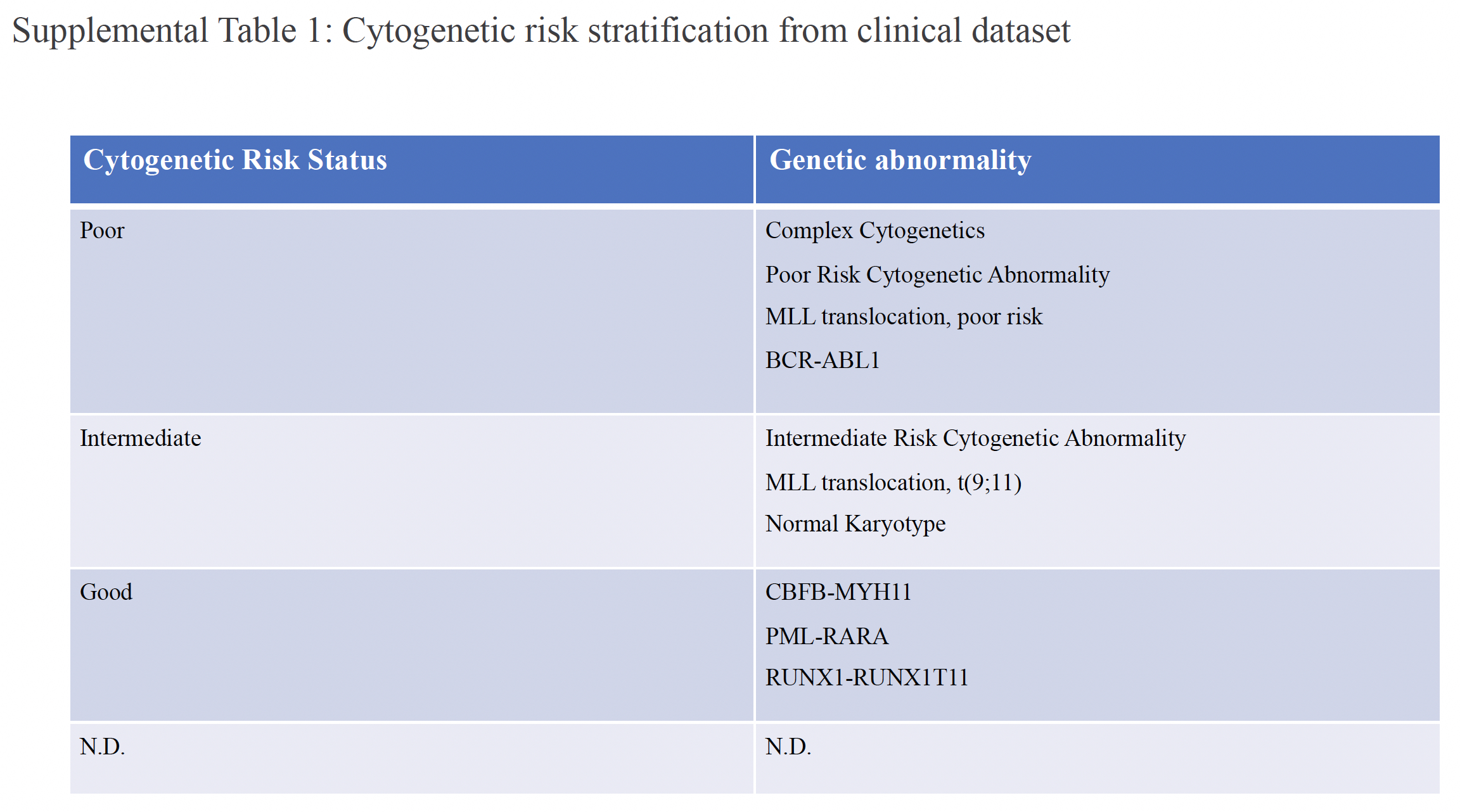

Supplement: Supplementary file 2 [file Image3.jpg]

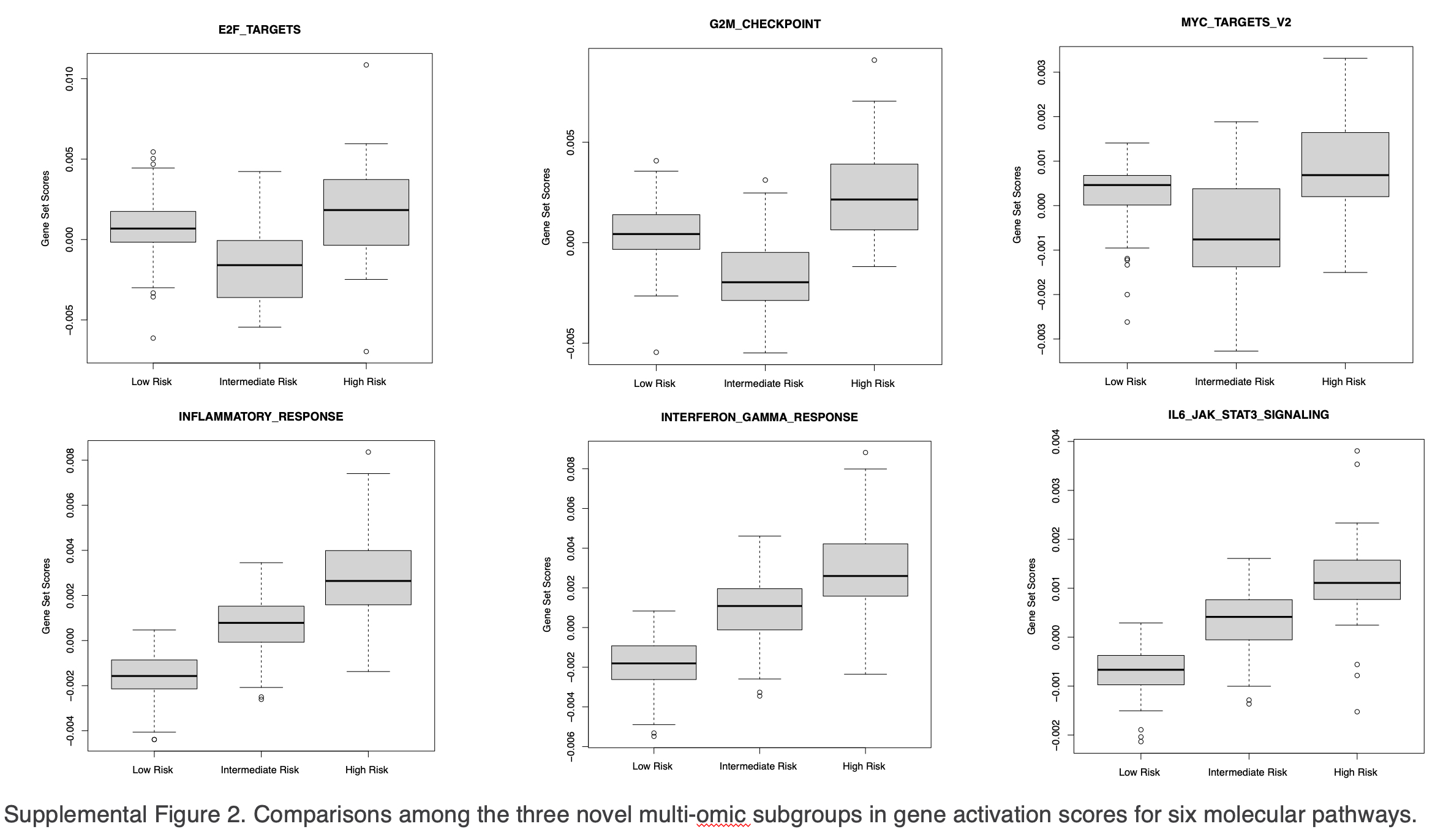

Supplement: Supplementary file 3 [file Image2.jpg]

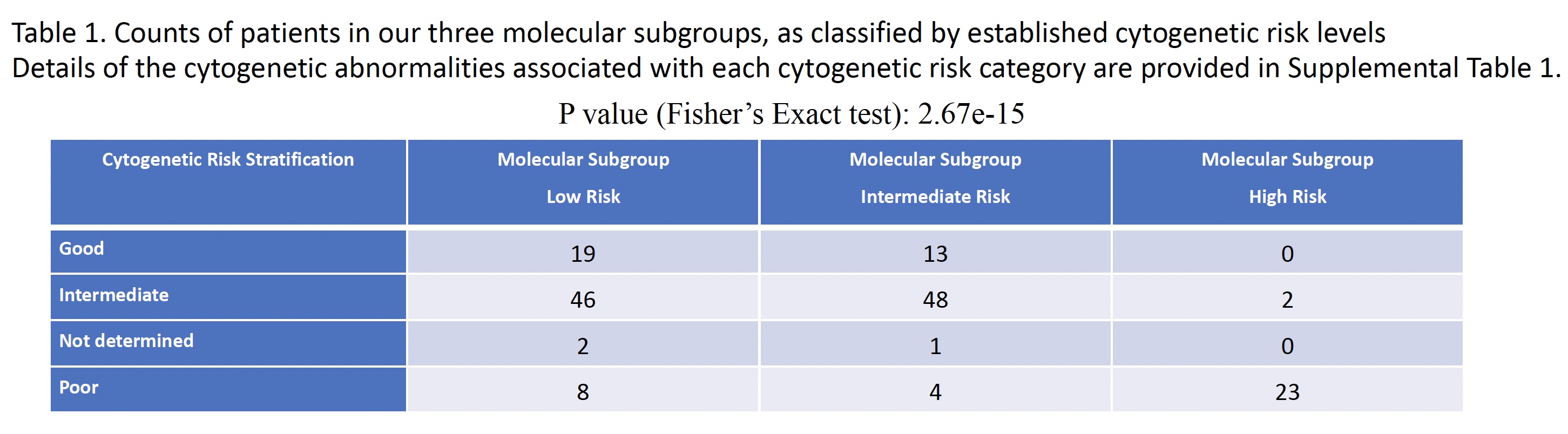

Supplement: Supplementary file 6 [file Image4.jpg]

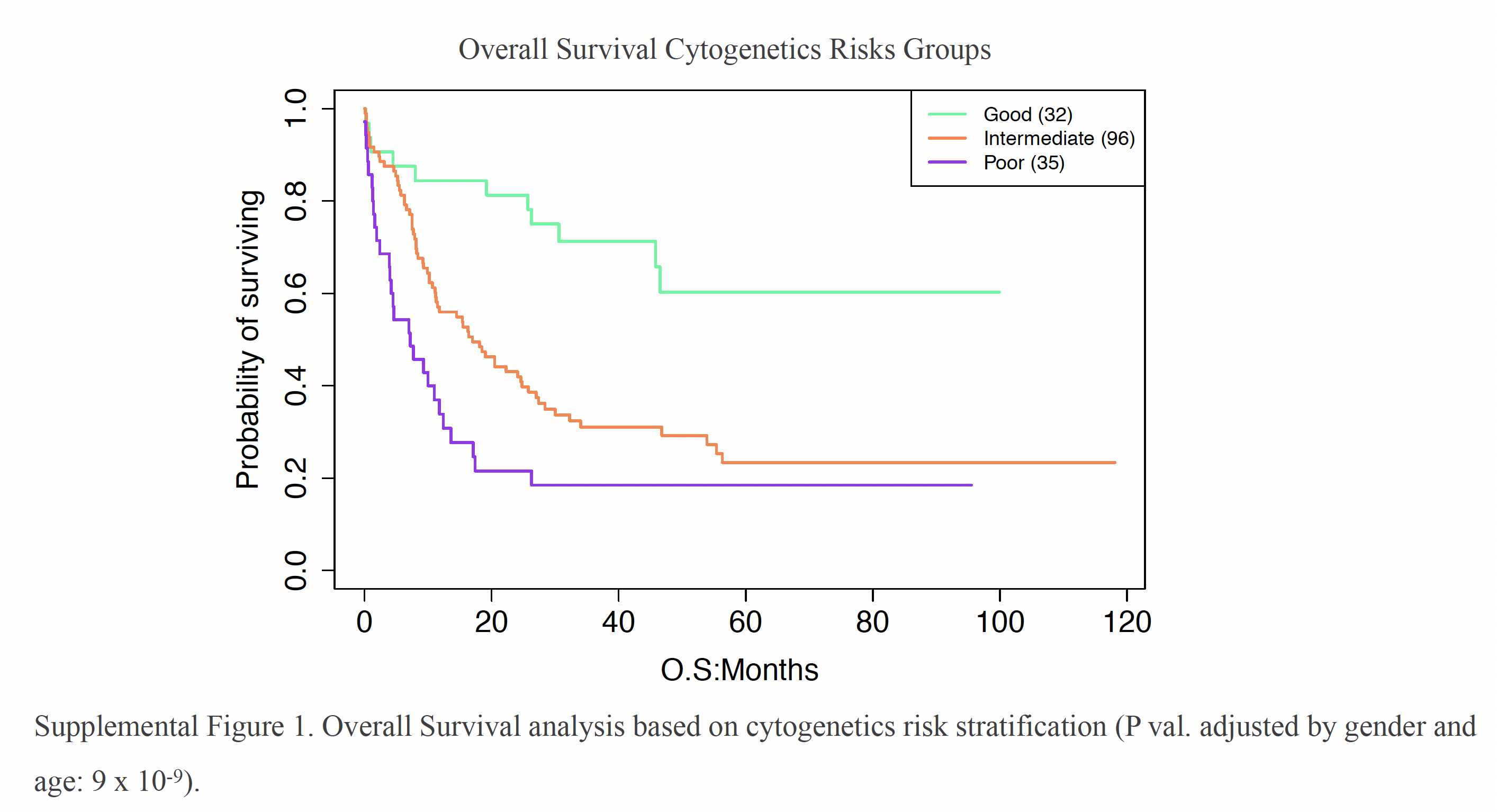

Supplement: Supplementary file 7 [file Image1.jpg]
